# Health situation in Germany during the COVID-19 pandemic. Developments over time for selected indicators of GEDA 2019/2020 – An update

**DOI:** 10.25646/9883

**Published:** 2022-07-06

**Authors:** Stefan Damerow, Alexander Rommel, Ann-Kristin Beyer, Ulfert Hapke, Anja Schienkiewitz, Anne Starker, Almut Richter, Jens Baumert, Judith Fuchs, Beate Gaertner, Stephan Müters, Johannes Lemcke, Jennifer Allen

**Affiliations:** Robert Koch Institute, Berlin Department of Epidemiology and Health Monitoring

**Keywords:** SARS-COV-2, BMI, SMOKING, DEPRESSION, HEALTH CARE UTILISATION, SUPPORT AT HOME

## Abstract

The spread of the coronavirus SARS-CoV-2 in 2020 and the containment measures associated therewith have changed many aspects of daily life. An impact on health even beyond infections itself is assumed as well. The health situation of the population in the first phase of the pandemic was thus analysed using data from the German Health Update (GEDA 2019/2020-EHIS). By continuing the survey, the analyses for 2020 are completed (n=26,507 participants), whereby the focus is now on the third phase of the pandemic (second wave of infection, gradual reintroduction of containment measures). The health indicators are presented on a monthly basis. As in the first phase of the pandemic, no pandemic-related changes were observed for tobacco smoking/ second-hand smoke exposure and for received/lack of/provided support. In contrast to the first phase of the pandemic, declines in utilisation of medical services and depressive symptoms are not observed in the third phase. The increase in body weight/body mass index after the first phase of the pandemic did not continue. The survey period allows for a comparison of the periods before and as of the pandemic situation. A decrease in the medical services utilisation and depressive symptoms as well as an increase in the body weight/body mass index is observed in the period from March 2020 to January 2021 compared to the pre-pandemic period from April 2019 to March 2020.

## 1. Introduction

The spread of the coronavirus SARS-CoV-2 in 2020 and the non-pharmaceutical containment measures associated therewith have changed many aspects of daily life in Germany. The SARS-CoV-2 infection situation in 2020 can be divided into three phases: Phase 1 with the first COVID-19 wave and the enactment of comprehensive containment measures (March to mid-May), Phase 2 (summer) with comparatively low infection incidence and less strict pandemic control measures (mid-May to September) and Phase 3 with the onset of the second COVID-19 infection wave and again gradual tightening of the containment measures (starting in October) and the peak of the second infection wave at the end of the year [1, 2]. There was fear that, in addition to infections, the containment measures as part of the pandemic could also have negative health consequences [3–5].

In 2020, the health situation of the population in the first two phases of the pandemic was analysed using the data from the German Health Update (GEDA 2019/2020-EHIS). The focus was on whether conspicuous changes in health status and health behaviour were observed from the beginning of the first COVID-19 wave [6]. Among the general population, no abnormalities were found in depressive symptoms, smoking behaviour, the use of support services by elderly people in the household, or the proportion of relatives providing care. Body weight and body mass index (BMI), in contrast, had slightly increased steadily on average among the population between March and September 2020. There was a temporary sharp decline in the use of general and specialist medical services during the first phase of the pandemic, but this appeared to have normalised in the second half of 2020.

The GEDA 2019/2020-EHIS survey ended just before the third phase of the pandemic, during which infection numbers again increased. Starting in November, a so-called nationwide ‘partial lockdown’ with contact restrictions was decided on and was tightened starting in mid-December 2020 [1]. To analyse possible effects, the survey was extended until the beginning of 2021 and now includes approximately 26,500 participants aged 15 years and above.

The currently available time series relating to the health situation with respect to the health indicators analysed in Damerow et al. 2020 [6] (depressive symptoms, smoking behaviour, medical services utilisation, BMI/body weight, as well as support services for older people) are updated in this paper. This will examine, whether the observed developments in the third phase of the pandemic continue or change as of October 2020. In addition to the presentation of time series, it will be analysed if there are differences compared to the periods before (April 2019 to first half of May 2020) and as of the onset of the pandemic situation (second half of March 2020 to January 2021).

**GEDA 2019/2020**

Fifth follow-up survey of the German Health Update

**Data holder:** Robert Koch Institute

**Objectives:** Provision of reliable information on the health status, health behaviour and health care of the population living in Germany, with the possibility of European comparisons

**Study design**: Cross-sectional telephone survey

**Population:** German-speaking population aged 15 and older living in private households that can be reached via landline or mobile phone

**Sampling:** Random sample of landline and mobile telephone numbers (dual-frame method) from the ADM sampling system (Arbeitskreis Deutscher Markt- und Sozialforschungsinstitute e.V.)

**Sample size:** 26,507 respondents

**Study period:** April 2019 to January 2021 (GEDA-EHIS to September 2020)

**GEDA survey waves:**

**►** GEDA 2009
**►** GEDA 2010
**►** GEDA 2012
**►** GEDA 2014/2015-EHIS
**►** GEDA 2019/2020

Further information in German is available at www.geda-studie.de

## 2. Methodology

### 2.1 Study design, sampling and weighting

#### Study design

The GEDA study is a nationwide cross-sectional survey of the adult population living in Germany. On behalf of the Federal Ministry of Health, the Robert Koch-Institute (RKI) has conducted the survey at intervals of several years since 2008 and the survey is part of the RKI health monitoring [7, 8]. GEDA captures a broad range of topics on health status, factors influencing the health situation, and the use of the health care system, as well as sociodemographic characteristics. The current GEDA survey was conducted as a telephone interview survey using a programmed, fully structured questionnaire (Computer Assisted Telephone Interview – CATI). It is based on a random sample of landline and mobile telephone numbers. The population comprises people aged 15 years or above living in private households, whose usual place of residence at the time of data collection is in Germany. Detailed information on the sampling design, sampling, weighting, and the indicators used in the GEDA 2019/2020-EHIS study are described in more detail elsewhere and will be discussed only briefly below [9, 10]. After the originally planned survey was conducted from April 2019 to mid-September 2020, a continuation of the data acquisition starting at the end of October 2020 was realised until January 2021, in order to observe the effects of the pandemic as it developed. The original study design was kept with a slightly shortened questionnaire. Until the survey was resumed, there was an interruption of the data collection of around six weeks between September and October 2020. No statements are thus possible for this period. The survey period from April 2019 to January 2021 with the shortened questionnaire will be referred to as GEDA 2019/2020, whereas GEDA 2019/2020-EHIS is to be understood as the detailed questionnaire until September 2020.

#### Sample

**Annex Figure 1:**
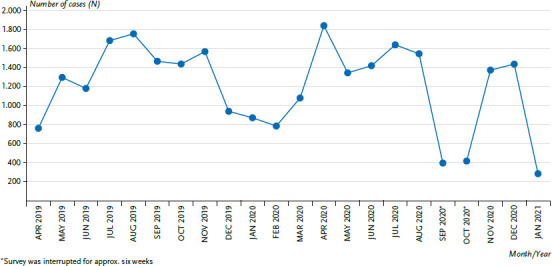
Development of the realised interviews in GEDA 2019/2020 over time, from April 2019 – January 2021 Source: GEDA 2019/2020

There were 23,001 respondents to the original data collection of GEDA 2019/2020-EHIS. The continuation between October 2020 and January 2021 includes 3,506 new participants. A total of 26,507 people (13,955 female, 12,552 male) thus participated in the cross-sectional study GEDA 2019/2020 between April 2019 and January 2021. On average, 1,205 (minimum: 282, maximum: 1,841) people were interviewed each month (Annex Figure 1).

#### Weighting

The weighting of the GEDA 2019/2020 sample comprises a design weighting (mobile and landline) and a so-called adjustment weighting to the official population figures, based on age, sex, federal state, and district type (as of December 31, 2018) as well as to the education distribution according to the International Standard Classification of Education (ISCED classification) in the microcensus (2017). The weighting process is described in detail elsewhere [9].

The probability of participation of certain population groups could furthermore be influenced due to the COVID-19 pandemic and the associated containment measures (for example home office, contact restrictions) [6]. For this reason, an additional adjustment weighting was made separately for the sampling periods before and as of the cut-off date of March 16, 2020 (adoption of the agreement between the federal government and federal states on guidelines to slow down the spread of the coronavirus [11]). Analogously to the adjustment weighting of the entire sample, the distributions for age, sex, and education were utilised thereby.

### 2.2 Indicators

The time series described in Damerow et al. 2020 [6] of selected indicators are updated in this paper. It was assumed that there could have been changes in the health status (especially mental health), health behaviour, health care and support services in the course of the pandemic. Methodologically, only those indicators were selected, which explicitly aimed at recoding facts at the time of the survey (e.g. referring to ‘currently’ in the wording of the questions).

#### Mental health

The presence of depressive symptoms was captured based on the self-reported data from the participants in the internationally established 8-item patient health questionnaire (PHQ-8) within the last two weeks [12]. The instrument comprises a total of eight domains of symptoms, which were designed and validated for the diagnosis of ‘major depression’, following the criteria established in the fourth edition of the Diagnostic and Statistical Manual of Mental Disorders (DSM-IV). The presence of depressive symptoms is assumed starting at a scale total value of at least ten of 24 points.

#### Body weight and body mass index

Respondents’ self-reported height was in cm and body weight in kg. The body mass index (BMI) is calculated as the ratio between body weight and height square (kg/m^2^).

#### Tobacco smoking and second-hand smoke exposure

The self-reported smoking status allows making a distinction between people, who currently smoke, and non-smokers. The daily exposure to second-hand smoke of current non-smokers was also acquired by means of self-reporting.

#### Utilisation of medical services

Dichotomous variables, which distinguish respondents who consulted a general practitioner or a specialist, respectively, from respondents without a corresponding utilisation, were formed from the self-reported data relating to the medical services utilisation in the last four weeks.

#### Received, lack of, and provided support

Based on the instrumental activities of daily life [13], people aged 55 and older (n=15,030) were asked about being able to perform seven different household activities (preparing meals, using the telephone, going shopping, managing medication, doing light housework, occasionally doing heavy housework, taking care of finances and everyday administrative tasks) each without help (details see [6]). If at least one activity causes some or large difficulties, questions were asked about receiving help, and the groups ‘received support’ and ‘did not receive support’ were formed. A lack of support was encoded for both groups, if, according to self-reporting, (more) help was required. Provided informal care or support at least once a week was recorded among all participants and was grouped as ‘support provided’ or ‘not provided’, respectively.

#### Education

Education levels according to the CASMIN classification (Comparative Analyses of Social Mobility in Industrial Nations) were used as indicator of social status. School and vocational qualifications serve to distinguish three groups with low, medium and high education level [14].

### 2.3 Statistical analyses

To update the time series, a statistical methodology is used, which is generally equivalent, but slightly changed, compared to the earlier analyses [6]. Using the survey procedures for complex samples and the weighting factors, three logistic regression models were estimated for dichotomous indicators and three linear regression models for metric indicators. Federal state, age, sex, education as well as the interaction between age, sex, and education were used as independent control variables. In the first model, an independent categorical variable is used to represent the monthly progression of indicators over the survey period. To smooth out the representation from Model 1, cubic splines were used based on the exact day of the interview date in order to model the monthly time course (in Damerow et al. 2020 the soothing was not implemented using a spline regression, but fourth order polynomials of the interview week).

The results of the first two model estimations were used to calculate adjusted predictions stratified by interview month. For the dichotomous indicators, the predictions can be interpreted as adjusted proportions (in %) and as adjusted mean values for metric indicators. The results are in each case presented in a graph, including a 95% confidence interval.

To quantify and to test a potential effect based on the pandemic situation, adjusted proportions/mean values were calculated for the periods before and as of the cut-off date of March 16, 2020 (agreement between the federal government and federal states on guidelines to slow down the spread of the coronavirus [11]) and were tested for statistical significance. For this, a third regression model was estimated with a binary variable for the differentiation of the periods. A statistically significant difference between the periods is assumed if the p-value of the binary variables is <0.05. The results of all indicators are summarized in Annex Table 1. To test for differential trends between the time periods with regard to sociodemographic variables, interactions with age, sex, and education group were tested. All analyses were conducted with StataSE 17.0 (Stata Corp., College Station, TX, USA, 2017).

## 3. Results

### 3.1 Mental health

**Figure 1:**
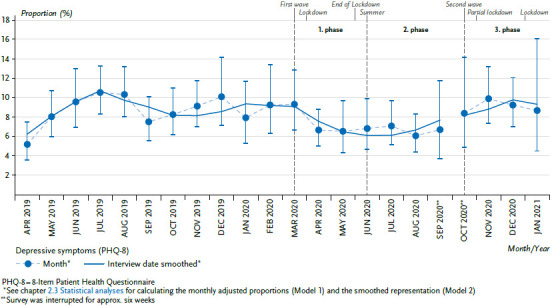
Depressive symptoms over time, from April 2019 – January 2021 (adjusted proportions) Source: GEDA 2019/2020

At the start of the pandemic situation and the associated containment measures, the adjusted proportions of people with depressive symptoms decreased initially, starting in April 2020, while they had increased in the reference period from April to August 2019 (Figure 1). The comparison of the periods before and as of March 16, 2020, likewise shows a statistically significant decrease from 8.9% to 7.6% (Annex Table 1). The interaction test for sex- or age-specific effects does not provide any relevant or significant differences, respectively (data not shown). However, the update of the development over time as of the onset of the third phase of the pandemic (October 2020 to January 2021) no longer shows any abnormalities compared to the previous year. As a whole, the progression thus indicates a temporary decrease of the depressive symptoms in the time from April to August 2020. The opposite progression in 2019 does not indicate that this is a returning seasonal effect in the summer of 2020.

### 3.2 Body weight and body mass index

**Figure 2:**
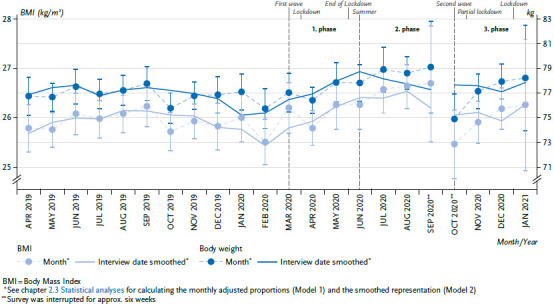
Body weight and body mass index over time, from April 2019 – January 2021 (adjusted mean values) Source: GEDA 2019/2020

Compared to the period April to August 2019, an increase of body weight as well as of BMI was observed in the period of the first two phases of the pandemic from April to August 2020 [6]. This increase of the monthly values did not continue in the third phase. Starting in October 2020, no substantial change in the mean adjusted body weight or BMI can be seen (Figure 2). The comparison between the periods before and as of March 16, 2020, results in a statistically significant increase in the mean adjusted body weight from 76.9 kg to 77.7 kg as well as in the BMI from 25.9 kg/m^2^ to 26.2 kg/m^2^. The interaction test with age and sex does not provide any relevant or significant differences, respectively (data not shown).

### 3.3 Tobacco Smoking and second-hand smoke exposure

**Figure 3:**
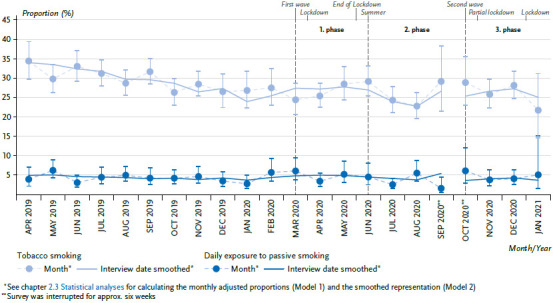
Tabacco smoking and second-hand smoke exposure over time, from April 2019 – January 2021 (adjusted proportions) Source: GEDA 2019/2020

As a whole, the analyses for current smoking in the period April 2019 to September 2020 prove a decrease of the proportions of tobacco smokers, but no noteworthy developments with the onset of the pandemic situation (March 2020) and the associated containment measures for the period until June 2020 (first phase of the pandemic) [6]. In the case of smoking, no clear or noteworthy trend, respectively, can be seen between the beginning of the third phase of the pandemic (October 2020) and January 2021 (Figure 3). Compared to the periods before and as of March 16, 2020, however, there is a significant decrease of the smokers in the population (29.4% and 26.2%).

For second-hand smoke exposure, there were no changes for the entire survey period. This is also confirmed by the comparison of the periods before and as of March 16, 2020: The adjusted proportion of people with a daily second-hand smoke exposure showed virtually identical results for both periods (4.4% and 4.3%).

In the case of both indicators, no age or sex effects can be detected based on the interaction test (data not shown).

### 3.4 Medical services utilisation

**Figure 4:**
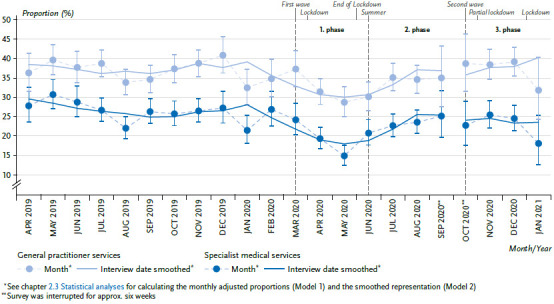
Outpatient medical services utilisation over time, from April 2019 – January 2021 (adjusted proportions) Source: GEDA 2019/2020

The results of the first analyses show a significant decline of the general and specialist medical services utilisation during the time of the containment measures between April and June 2020 (first phase of the pandemic). However, the medical services utilisation has quickly normalised to pre-pandemic level again [6]. This development is confirmed by the new data for the months of October 2020 (start of the third phase of the pandemic) to January 2021. After October 2020, the medical services utilisation did not decline as pandemic containment measures increasingly resumed, but remained at a relatively constant level (Figure 4). In the case of the estimated adjusted proportions, the comparison of the periods before and after March 16, 2020, reflects significant differences in general (36.9% to 34.6%) and in specialist (26.3% to 22.0%) medical services. A statistically significant sex effect is reflected in that the decline in general medical services utilisation occurs exclusively among women. In the case of the specialist medical services, there is also an age effect, which relates to 80-year-olds and above. This age group is the only one, in which a constant utilisation is observed (data not shown).

### 3.5 Received, lack of, and provided support

**Figure 5:**
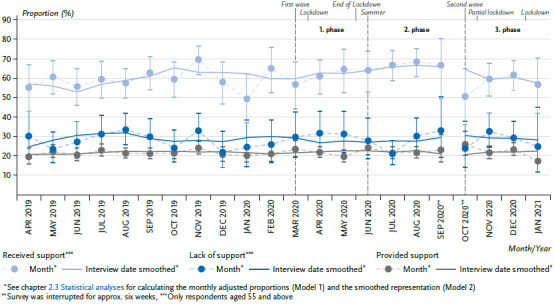
Received, lack of, and provided support over time, from April 2019 – January 2021 (adjusted proportions) Source: GEDA 2019/2020

There are no pandemic-related abnormalities for the adjusted proportions of the indicators relating to the support services in the household for older people in the period between April 2019 and September 2020 [6]. Further temporal trends between the start of the third phase of the pandemic from October 2020 to January 2021 also do not show noteworthy trends in the case of any of the three indicators (Figure 5). With 59.9%, the adjusted proportion of people 55 years or older who received support with household activities in the pre-pandemic period until March 16, 2020, remained slightly lower than in the comparison period (62.6%, difference not statistically significant). The estimate of a lack of support (aged 55 years or above) likewise remained virtually identical (29.0% vs. 28.4%) as did the adjusted proportions of people who provided support (21.6% vs. 22.2%).

No age or gender effects are detectable for any of the three indicators based on the interaction test (data not shown).

### 3.6 Differences by education

The trend tests conducted for the indicators observed here for the comparison of the observed periods before and after March 16, 2020, reflect only a few noteworthy interaction effects with the respondents’ education. The trends predominantly do not systematically differ for the different education groups. The medical services utilisation is an exception. Significant differences according to the education level are reflected here insofar as the utilisation declined more strongly in the higher and lower education group than in the medium education group (data not shown). A smaller decline of the utilisation of general practitioners in the medium education group for the period until December 2020 [6] cannot be determined any longer for the currently observed period.

## 4. Discussion

Using the data from GEDA 2019/2020-EHIS, a differentiated picture with regard to potential effects due to the pandemic situation and the associated non-pharmaceutical intervention measures for containing the COVID-19 pandemic was reflected in the survey period April 2019 to September 2020 in the case of selected health indicators [6]. The extension of the data collection from October 2020 to January 2021 completes the time series for 2020. The proportion of smokers, people with second-hand smoke exposure, as well as the received, lack of, and provided support is still inconspicuous even with the onset of the third phase of the pandemic until the end of 2020. There are no changes, which appear to be related to the pandemic situation. No further increase in BMI or body weight is observed for the months October 2020 to January 2021, but rather a relatively constant level. No change is observed in the case of the medical services utilisation, for which a new decline could have been expected for the third phase of the pandemic due to the second wave of infection and the reintroduction of containment measures as of October 2020. The same applies to the estimated proportion of people with depressive symptoms, which appears to be at pre-pandemic levels with the onset of the third phase of the pandemic and the new gradual tightening of containment measures.

The study GEDA 2019/2020 provides data covering the period from approximately one year before the onset of the pandemic situation as well as approximately the entire year 2020. It is thus possible to analyse effects of the COVID-19 pandemic with regard to the health situation beyond infections situation. A special feature of the study are the standardised survey instruments, which are not COVID-19-specific, but were used identically before and as of the beginning of the pandemic situation and thus do not capture potential effects of the pandemic retrospectively. The authors are not aware of any Germany-wide, population-based health study that provides comparable data. The GEDA 2019/2020-EHIS study was originally not designed for this purpose [9], which is why the selection of the indicators is limited to the existing database, and thus only reflects a section of possible effects on the health and the health behaviour. Due to the fact that GEDA 2019/2020 was continued with identical methodology, the well-known limitations of telephone studies and specific potential limitations of the present analyses still apply [6, 9]. The results for September and October 2020 as well as January 2021 should be interpreted carefully due to the low number of cases (Annex Figure 1) and the higher statistical uncertainty associated therewith. Due to the interruption of the survey for approximately six weeks in September and October, the survey points additionally do not directly merge into one another.

Initial fears among the population that depressive symptoms could increase due to the COVID-19 pandemic or due to the containment measures, are not supported by the presented results. A slight decline as part of the first phase of the pandemic can even be observed. The update of the time series as of the beginning of the third phase of the pandemic (October 2020 to January 2021) shows that this is a temporary decline of depressive symptoms. Additional analyses relating to single items already indicated in the previous work that the decline particularly affected the areas of fatigue, loss of energy, and concentration difficulties [6]. These occur as side effects of chronic stress [15]. A recent review of mental health of the adult population in Germany during the COVID-19 pandemic, based primarily on data from the first and second phase of the pandemic, also found no evidence of serious mental health impairment in population [16]. Intra-individual deteriorations as well as improvements of mental health were observed in a few, non-representative longitudinal studies [16]. The analyses reported here, which include the further progression of the pandemic until the beginning of 2021, do not suggest that mental stress has a negative cumulative effect over time. This result is also consistent with available data on suicide rates. Fears expressed in advance of increased suicide rates during the pandemic [17] could not be confirmed. In 2020, the deaths due to intentional self-harm are at a comparable level as in previous years [18]. These results hold true for the general population, there is an additional need for research here with regard to changes in specific, vulnerable population groups [16].

In consideration of the months of October 2020 to January 2021, there is no further increase in body weight and BMI, nevertheless, compared to the pre-pandemic period, mean BMI has increased by 0.3 units and body weight by 0.8 kg. Measures for containing the COVID-19 pandemic have resulted in changes in daily life that have favoured an increase in body weight. A review that compiled risk factors for weight gain shows that the body weight among people who specify having gained weight during a quarantine, increases between 0.5 and 1.8 kg (±2.8 kg) after only two months. More sedentary activities and less physical activity, more frequent snacking (especially after dinner), increased consumption of alcohol, and reduced consumption of water, more frequent emotionally motivated eating, reduced sleep quality and the presence of overweight or obesity, are often mentioned as risk factors [19]. Having overweight or obesity, in turn, are risk factors for many health problems, and thus have a large public health relevance. Persistent weight gain could promote the occurrence of health problems and diseases associated with overweight and obesity. At population level, it has been shown that mean body weight and BMI have not decreased again as the pandemic has progressed. It will be evaluated in following analyses, to what extent body weight and BMI will permanently remain at that level, and how changes are distributed in different groups.

Even though the available data show a decrease of the proportions of smokers in the survey period, they do not reflect an influence of the COVID-19 pandemic or of the pandemic-related measures. This is generally plausible in light of the long-term decrease in the smoking behaviour in Germany [20]. Due to the fact that smoking is considered to be a risk factor for a severe COVID-19 progression [21], a decrease of the smoking prevalence would have been expected. In light of the fact that (increased) smoking is used as strategy to manage feelings, such as anxiety and worries [22, 23], an increase in the smoking prevalence would also have been plausible. Results of other studies prove especially an increase in the strength of tobacco consumption [23, 24], which was not subject matter of the present analysis, however. For the daily second-hand smoke exposure, no change was reflected in the progression of the pandemic, even though a decrease could also have been expected here, at least in accordance with the smoke prevalence.

As a result of the containment measures taken during the first phase of the pandemic, the population obviously dispensed with outpatient medical services utilisation in a distinct way. Similar developments were also shown nationally and internationally in other studies for various service areas [25–31]. However, a new decline at the onset of the third phase of the pandemic starting in October 2020 was not observed with the present data. However, there is also no strong increase that could indicate a catch-up effect in the use of medical services. The population thus adjusted its behaviour for seeking help to the pandemic situation insofar as outpatient services were utilised significantly more frequently than during the first phase of the containment measures in the spring. As a whole, the partially sharp decline between April and June 2020 leads to a decrease of the frequency of medical contacts in the entire reference period before and as of the pandemic situation. Evaluations of the case numbers of the SHI-accredited care also show that the level of utilisation from the year 2019 had not been reached yet in the fall of 2020 [32]. It is also noticeable in the present analyses that the decline of the general medical utilisation is found especially among women. General medical practices are often also visited for preventative purposes, whereas many specialist medical practices are visited more because of concrete treatment reasons. Due to the fact that women tend to use preventative offers more strongly than men, the decline could be caused by a temporary postponement of such services. The decrease in utilization was not found among older respondents. Due to chronic diseases, they are dependent more strongly on doctor’s visits than younger people and are possibly less likely to postpone treatments.

For the population aged 55 and older with difficulties in daily household activities, the results show that there was obviously sufficient support for household activities during the measures for containing the COVID-19 pandemic. This suggests that family and neighbourhood networks or even professional support were in fact sufficiently available and were used in 2020.

The data from GEDA 2019/2020 makes it possible to analyse the health situation of the population for the entire year of 2020 in light of the COVID-19 pandemic and to draw comparisons with the pre-pandemic time. Based on the analyses presented in 2020 [6], a large degree of stabilisation is observed. Indicators, which did not show abnormalities in the first two phases of the pandemic, also did not change as of the third phase with the onset of the second wave of infection and the containment measures, which were gradually tightened again. In the case of indicators with pandemic-related effects initial phase of the pandemic, such as the BMI/body weight or the medical services utilisation, similar changes cannot be detected between October 2020 and January 2021. The current results apply only to the general population. Different population groups may have been affected differently to the challenges and stresses in the pandemic. Future research should focus on further population groups, such as people with low income, unemployed people, single parents, the elderly, or people with chronic diseases, such as diabetes [33]. With regard to differences in education, the analyses conducted here focus exclusively on different trends for the reference periods before and after March 16, 2020. These trends could only been shown for the indicator of the utilisation. The analyses take into account whether there were significant differences in the prevalence differences in the two reference periods with respect to the observed education groups (absolute differences). Relative differences between the education groups or differences in the prevalence ratio, respectively, are not considered. They could also be observed when the prevalence increases or decreases equally in all comparison groups. For example, if prevalence in the highest and the lowest education group decreases equally by ten percentage points from 40% and 20% to 30% and 10%, respectively, in the reference period, then the prevalence differences between the education groups do not change, but the prevalence ratio between both groups does.

At the end of data collection for the GEDA 2019/2020 study, the infection incidence of the second wave had not yet subsided and more extensive containment measures were subsequently in place [1, 2]. Consequently, no final conclusion can be drawn for the entire pandemic and the associated containment measures. For this purpose, it would be necessary to continuously monitor the development of the health situation of the population during the further progression of the pandemic.

## Key statement

- The frequency of depressive symptoms has decreased only temporarily in the first phase of the pandemic.
- The initial increase of the body weight in the first phase of the pandemic has not continued.
- There are no indications towards changes in smoking behaviour due to the pandemic situation.
- A significant decrease of the utilisation of general and specialist medical services, as in the first phase of the pandemic, is not observed in the third phase.
- The proportion of people with support for household activities has remained largely constant during the entire observation period.

## Appendix

**Annex Table 1:** Comparison of adjusted proportions and mean values before and after March 16, 2020 Source: GEDA 2019/2020

Indicator	Adjusted proportion/mean value with 95% CI (before March 16, 2020)	Adjusted proportion/mean value with 95% CI (as of March 16, 2020)	Number of cases (GEDA 2019/2020 overall)
Utilisation: General practitioners (%)	36.9 (35.8–38.1)	**34.6** (33.3–35.9)	26,427
Utilisation: Specialist (%)	26.3 (25.2–27.3)	**22.0** (21.0–23.1)	26,379
Mental health: Depressive symptoms (PHQ-8) (%)	8.9 (8.2–9.8)	**7.6** (6.8–8.5)	25,981
Received support in the household (%)^*^	59.9 (57.0–62.8)	62.6 (59.5–65.6)	4,504
Required support in the household (%)^*^	29.0 (26.2–31.9)	28.4 (25.3–31.6)	4,488
Provided support (%)	21.6 (20.6–22.6)	22.2 (21.1–23.4)	26,479
Tabacco smoking (%)	29.4 (28.3–30.5)	**26.2** (24.9–27.5)	26,497
Daily second-hand smoke exposure (%)	4.4 (3.8–5.1)	4.3 (3.6–5.1)	20,878
Body mass index	25.9 (25.8–26.1)	**26.2** (26.0–26.3)	26,128
Body weight	76.9 (76.5–77.3)	**77.7** (77.2–78.2)	26,160

CI = Confidence interval, PHQ-8 = 8-item patient health questionnaire, bold = p-value <0.05

^*^ Only participants ≥55 years of age
